# The effect of acetate Ringer’s solution versus lactate Ringer’s solution on acid base physiology in infants with biliary atresia

**DOI:** 10.1186/s12887-021-03074-4

**Published:** 2021-12-20

**Authors:** Xiang Liu, Hongyan Cao, Xiaona Tan, Jing Shi, Li Qiao, Qi Zhang, Lei Shi

**Affiliations:** 1 Department of Operating Room and Anesthesiology, The children’s hospital of hebei province, No. 133 Jian Hua South Road, Shi Jiazhuang, 050030 China; 2 Department of Neurological Rehabilitation, The children’s hospital of hebei province, No. 133 Jian Hua South Road, Shi Jiazhuang, 050030 China

**Keywords:** Sodium acetate, Acid-base balance, Lactate, Children

## Abstract

**Background:**

The choice of the perioperative crystalloid is a key component of the fluid management and must take into account the liver function and the appearing metabolic disorders to avoid increase the liver extra metabolism. The aim of this study is to analyze the effect of acetate Ringer’s solution or lactate Ringer’s solution in biliary atresia patients.

**Methods:**

We included 68 infant patients aged between 21 ~ 65 d, ASA physical status II or III, who underwent elective Kasai hepatoportoenterostomy, received either AR and LR for intravenous fluid resuscitation according to their group allocation. Lactate concentration, serum electrolytes and pH were noteded before skin incision (T_1_), end of surgery (T_2_) and postoperative 12 h. We also recorded the time of operation, stay of hospital, loss of blood and urinary, total volume of infusion of crystalloid.

**Results:**

Lactate level was significantly higher in Group LR than in Group AR patients at T_2_ (0.76 ± 0.13 versus 0.57 ± 0.22, *P* = 0.03). Compared with T_3_, sodium and chlorine were significantly higher in two groups at T_2_ (145.2 ± 3.1 versus 143.4 ± 3.4 and 104.6 ± 3.7 versus 105.2 ± 2.1). No significant differences were noted in potassium, HCO_3_^−^ and calcium. There was no statistically significant difference in pH. No glycopenia was recorded in two groups. No significant difference was noted in administration of vasoactive drug (0.7% versus 1%).

**Conclusions:**

Resuscitation with AR and LR was associated with similar clinical improvement in infants with biliary atresia. Use of AR reduced the level of lactate comparison with LR.

## Introduction

The aim of perioperative fluid therapy is to provide adequate intravascular volume to ensure tissue perfusion and cellular oxygenation that is the physiologic goal independent of the type of surgery. Biliary atresia is a neonatal liver disease characterized by progressive obstruction and fibrosis of the extrahepatic biliary tree as well as fibrosis and inflammation of the liver parenchyma [1]. The current treatment for biliary atresia involves sequential surgical intervention with the Kasai hepatoportoenterostomy [2]. The great challenge is that multiple factors affect the perioperative fluid management that may change the acid base balance and electrolyte in Biliary atresia. The choice of the perioperative crystalloid is a key component of the fluid management and must take into account the liver function and the appearing metabolic disorders to avoid increase the liver extra metabolism. The World Health Organization advocated lactate Ringer’s solution (LR) as the preferred fluid for correction of severe diarrheal dehydration [3]. The compositional properties have continued to sustain lactate Ringer’s solution as the seemingly ideal resuscitation fluid. Recent critical review has nevertheless brought lactate Ringer’s use into question, such as hepatic mediated metabolism, and increased aerobic demand [4].

Acetated Ringer’s solution (AR) do not display these shortcomings. Acetate as an alternative anion has been proposed [5]. Its advantages over standard lactate include its aqueous solubility, inert bioactivity and smaller molecular weight. The metabolism of lactate is dependent on the kidney and liver. Unlike lactate, acetate is also more rapidly metabolised with less oxygen demand and extra hepatic [6]. Therefore it could reduce the liver metabolic burden, especially in infants who already had liver function damage. We hypothesized that liver relatively had a better functional reserve to metabolize the extra lactate after acetate Ringer infusion than that after lactate Ringer’s solution. To date, and to our knowledge, there was a paucity of evidence in the literature on comparative influence of acetate Ringer’s solution or lactate Ringer’s solution. The primary main objective of this randomized controlled trial was to compare the effect of acetate Ringer’s solution or lactate Ringer’s solution on lactate and HCO_3_^−^ level in biliary atresia patients. The secondary objectives included determination of arterial pH, electrolytes, body temperature, urine volume, use of vasoactive drugs and hypoglycemia.

## Methods

### Study design

This randomized, double-blind controlled trial was approved by the Hospital Ethics Committee (Hebei Medical University affiliated Children’s Hospital of Hebei Province, 2,019,103) and was registered with ClinicalTrials (ChiCTR2000041129). Written informed consent was obtained from the parents of all patients during the preoperative visit. We included 68 infant patients aged between 21 ~ 65 d, ASA physical status II or III, who underwent elective Kasai hepatoportoenterostomy. Inclusion criteria were contained to receive treatments, no respiratory distress syndrome and renal failure. Exclusion criteria were fever, abnormal coagulation function, anemia, complicated congenital heart disease requiring emergency cardiac surgery. We had a preoperative liver protection therapy and maximally decreased the alanine transaminase and aspartate aminotransferase concentrations. We randomly assigned eligible patients using computer generated variable block randomization, concealed by sequentially assigned sealed randomization envelopes, to receive either LR and AR. Sequence generation and preparation of randomization envelopes were completed by Q.Z. independent of the research team and who had no further role in the trial. Envelopes were opened immediately before induction of anesthesia by L.Q. who was aware of the randomization outcome only after the envelope was opened.

### Anesthesia management

The patient was placed on a thermostatically controlled warming blanket and monitoring attached (electrocardiography, noninvasive blood pressure, pulse oximetry and P_ET_CO_2)_ prior to induction. Invasive blood pressure and central venous pressure were implemented after endotracheal intubation. Hemodynamics was monitored using a device (Mostcare care, Italy) via arterial catheterization. The core temperature was monitored continuously with the probe which was set on the pharynx nasalis. General anesthesia was induced by intravenous administration of propofol 1 to 2 mg/kg and fentanyl 2 ~ 3 μg/ kg. Neuromuscular blockade was produced by cisatracurium. Patients were intubated with a plain endotracheal tube (appropriate size 3.5 or 4.0). Anesthesia was maintained with inhalation of 0.5% ~ 1% sevoflurane in oxygen and air (FiO_2_ 45%) and remifentanil 0.25 ~ 0.3 μg/kg/min. Controlled mandatory ventilation (Dräeger Fabius, Germany) with oxygen-air mixture was used with a programmed inspiratory tidal volume of 8 to 10 ml/kg. P_ET_CO_2_ was maintained between 35 and 45 mmHg by adjusting the respiratory rate. The ventilation was delivered with a fixed inspiratory to expiratory ratio of 1:1.5. Respiratory parameters were adjusted or medicated according to the results of blood gas analysis.

### Fluid therapy

Patients received either LR or AR for intravenous fluid resuscitation according to their group allocation. Holliday and Segar protocol is still widely used as the most common formula to calculate fluid volume in the intravenous period. According to this calculation, the amount of fluid is given is 4 ml/kg for the first 10 kg of the child, 2 ml/kg in addition to 40 ml and 1 ml/kg in addition to 60 ml. The 3rd space loss is roughly estimated as 2 ml/kg/h for superficial surgery, 4 ~ 7 ml/kg/h for thoracotomy and 5 ~ 10 ml/kg/h for abdominal surgery [7]. Arterial blood was then collected every 1 h until the end of surgery. Trigger for blood transfusion depends on age, hemoglobin level and associated disease state. Based on the patient blood management programs restrictive hemoglobin thresholds may be indicated in infants and children (target of 7 g/dL for haemodynamically stable patient). The hemoglobin was measured and blood transfusion was determined in time to ensure adequate oxygen supply. The choice of specific agents and interventions was implemented to the discretion of the attending physician specialists.

### Data collection

Blood gas analysis was performed before skin incision (T_1_), end of surgery (T_2)_ and postoperative 12 h (T_3)._ A standard blood gas analyzer (ABL 800 FLEX, Denmark) was used. The blood glucose concentration was detected and the incidence of perioperative hypoglycemia (blood glucose <2.8 mmol/L) was recorded. The glucose solution was injected reasonably to treat hypoglycemia. Lactate concentration, serum electrolytes, and pH of the patients were documented from blood gas. During the anesthesia, we also recorded the time of operation, stay of hospital, loss of blood and urinary, red blood cell transfusions and total volume of infusion of crystalloid. Intraoperative body temperature was monitored, and intraoperative vasoactive agents and hypoglycemia were recorded.

### Statistical analysis

All data were recorded using a standardized data collection sheet and analyzed using the statistical software SPSS Statistics 18 (SPSS Inc., Chicago, IL, USA). On the basis of the results of a previous study, we assumed that the difference between the groups with respect to the primary outcome of lacetate concentration would be 0.12 mmol/L with a standard deviation of sample difference 0.36, and thus, 32 patients were required per sequence to achieve the desired power of 90% (β = 0.10) at the 5% (α = 0.05) level of significance. Based on this configuration, the study was designed to enroll 68 patients. The Shapiro-Wilk test was used to test for normality. Because most of the data were normally distributed, they are presented as mean ± standard deviation. Continuous data, if normally distributed, were compared by Student *t* test and, if abnormally distributed, were compared by Friedman test to describe changes of measurement parameters within a group during the course of time (three points of measurement). In order to compare differences between the groups an ANOVA was followed by a Student *t* test. Nonparametric variables were compared between the groups using χ2 test (Fisher exact test if cell frequencies were small). All tests were 2-tailed, and a *P* value < 0.05 was considered as significant.

## Results

**Fig. 1:**
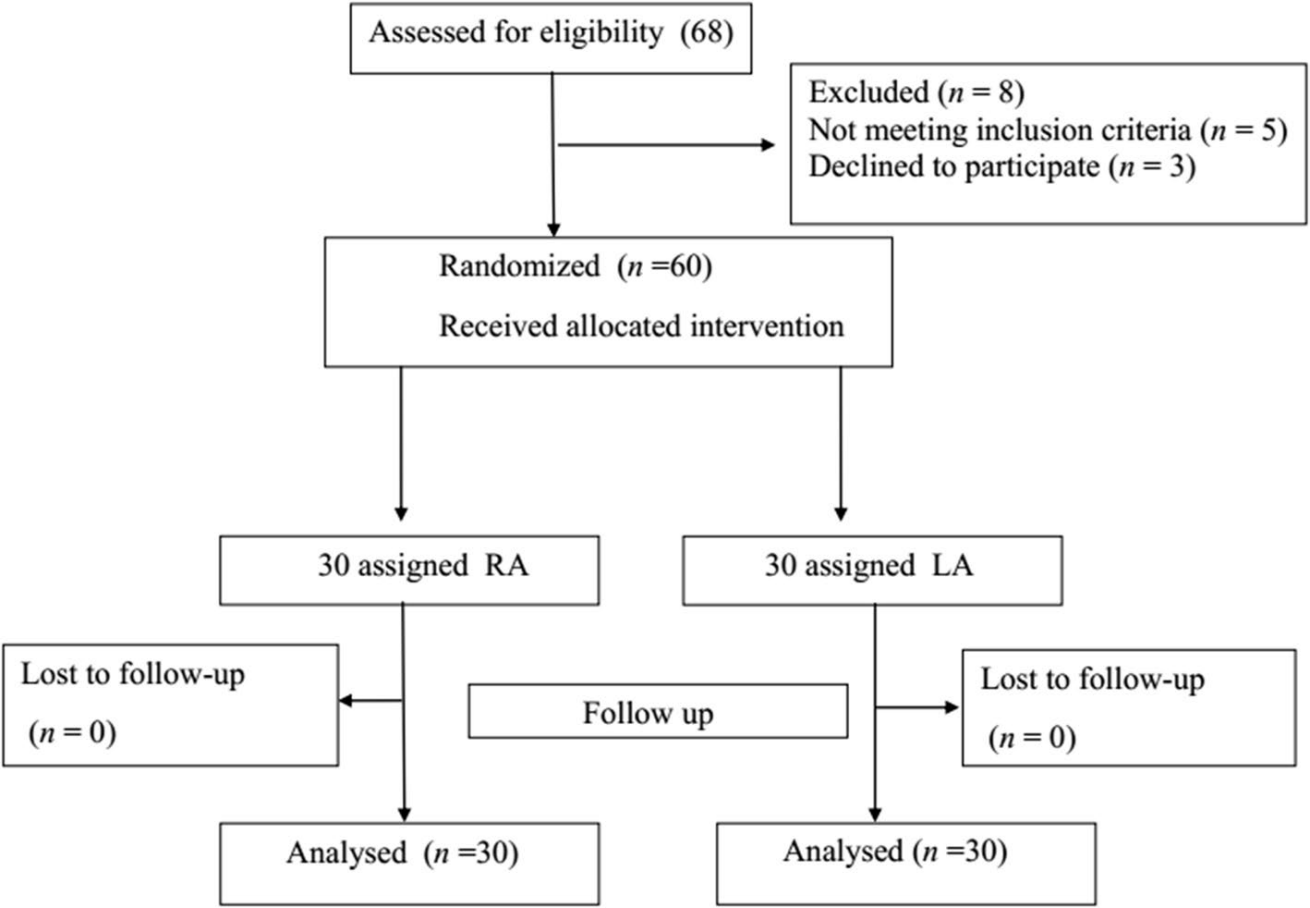
Consort flow chart. A total of 68 children were enrolled and completed the study

During the study period, 68 children were assessed, and 60 were randomized (Fig. 1). The two groups had comparable demographic characters such as age, weight, sex, time of operation, stay of hospital, loss of blood, red blood cell transfusions, urinary and total volume of infusion of crystalloid. We did not notice significant differences concerning patients’ demographic data (Table 1). There were also no significant differences between the groups in coagulation function (PT, Prothrombin time; APTT, Activated partial thromboplastin time) and hepatic function (ALT, Alanine transaminase; AST, Aspartate aminotransferase) (Table 2).

**Table 1:** Atient characteristics and the comparative data between the two groups

	Group LR	Group AR	*P* value
Male/female	21/9	16/14	0.288
Age (*d*)	34 ± 11	33 ± 14	0.780
Weight (*kg*)	5.3 ± 1.4	5.1 ± 1.7	0.748
Crystalloid Fluid (*ml*)	265 ± 45	245 ± 67	0.616
Urine quantity (*ml*)	52 ± 20	43 ± 16	0.482
Blood loss (*ml*)	78 ± 14	74 ± 25	0.325
Duration surgery (*h*)	6.8 ± 0.7	6.6 ± 0.5	0.403
Red blood cell transfusions (*ml*)	65 ± 14	73 ± 25	0.788
Hospital stays (*d*)	11.7 ± 1.6	10.7 ± 1.8	0.065
Vasoactive agents (*n*)	2 (6.7%)	3 (5%)	0.99
Hypoglycemia (*n*)	0	0	–

*Note*: Data are given as mean ± SD or number (percentage)

**Table 2:** Coagulation function and hepatic function were compared in two groups

	Group LR	Group AR	*P* value
ALT (*U/L*)	102 ± 12	97 ± 25	0.533
AST (*U/L*)	86 ± 38	75 ± 24	0.472
APTT (*S*)	32.1 ± 4.5	31.1 ± 4.7	0.393
PT (*S)*	11.3 ± 0.8	10.1 ± 0.7	0.375

*Note*: Data are given as mean ± SD

*ALT* Alanine transaminase, *AST* Aspartate aminotransferase, *APTT* Activated partial thromboplastin time, *PT* Prothrombin time

Lactate and serum electrolytes levels were compared between the two groups at different time points. Lactate level was significantly higher in Group LR than in Group AR patients at T_2_ (0.76 ± 0.13 versus 0.57 ± 0.22, *P* = 0.03) (Table 4). Compared with T_3_, sodium and chlorine were significantly higher in two groups at T_2_ (145.2 ± 3.1 versus 143.4 ± 3.4 and 104.6 ± 3.7 versus 105.2 ± 2.1) (Table 3). No significant differences were noted in potassium, HCO_3_^−^ and calcium. Temperature was significantly higher at T_3_ in two groups (37.2 ± 0.31 and 37.3 ± 0.37). There was no statistically significant difference in pH (Table 4).

**Table 3:** Electrolytes levels were compared between the two groups at different time points

Time	LR	AR	*P* value
	Na^+^ (*mmol/L*)		
T_1_	140.3 ± 2.6	142.5 ± 2.7	0.129
T_2_	145.2 ± 3.1^a^	143.4 ± 3.4 ^a^	0.203
T_3_	139.2 ± 4.9	138.5 ± 2.3	0.734
	K^+^ (*mmol/L*)		
T_1_	4.01 ± 0.43	3.89 ± 0.47	0.587
T_2_	4.06 ± 0.23	3.81 ± 0.41	0.111
T_3_	3.75 ± 0.32	4.03 ± 0.27	0.055
	Ca^2+^ (*mmol/L*)		
T_1_	1.18 ± 0.07	1.24 ± 0.07	0.136
T_2_	1.20 ± 0.03	1.22 ± 0.06	0.495
T_3_	1.17 ± 0.06	1.19 ± 0.05	0.507
	Cl^—^ (*mmol/L*)		
T_1_	102.4 ± 3.7	104.1 ± 2.1	0.212
T_2_	104.6 ± 3.2^b^	106.4 ± 2.0^b^	0.753
T_3_	100.7 ± 1.8	101.4 ± 2.9	0.617

*Note*: Data are given as mean ± SD

^a,b^ *P* < 0.05 versus T_1_ and T_3_ in Na^+^ and Cl^—^

**Table 4:** Temperature, pH, HCO3^—^ and lactate levels were compared between the two groups at different time points

Time	LR	AR	*P* value
	Temperature (*°C*)		
T_1_	36.8 ± 0.23	36.8 ± 0.25	0.625
T_2_	36.8 ± 0.17	36.9 ± 0.27	0.093
T_3_	37.2 ± 0.31*	37.3 ± 0.37*	0.746
	pH		
T_1_	7.37 ± 0.05	7.38 ± 0.05	0.790
T_2_	7.40 ± 0.04	7.37 ± 0.06	0.207
T_3_	7.40 ± 0.05	7.39 ± 0.04	0.476
	HCO_3_^—^ (*mmol/L*)		
T_1_	22.4 ± 2.0	21.5 ± 2.4	0.707
T_2_	20.1 ± 1.8	20.9 ± 3.1	0.796
T_3_	22.1 ± 1.6	21.9 ± 1.1	0.743
	Lactate (*mmol/L*)		
T_1_	1.09 ± 0.34	0.92 ± 0.38	0.343
T_2_	0.76 ± 0.13^#^	0.57 ± 0.22	0.030
T_3_	1.05 ± 0.25	0.92 ± 0.21	0.229

*Note*: Data are given as mean ± SD

*P < 0.05 versus T_1_ and T_2_ in temperature

^#^ *P* < 0.05 versus AR in lactate

No glycopenia was recorded in the two groups. No significant difference was noted in administration of vasoactive drug (0.7% versus 1%) (Table 1).

## Discussion

The main findings of this study were the demonstration that acetate Ringer’s solution was safe as a resuscitation medium, and further, that it might have some clinical advantages when compared to lactate Ringer’s solution as a control group.

Administration of intravenous solutions required to correct physiological functions that have been altered due to surgical stress and anesthetic agents and to maintain body homeostasis to provide oxygen to the tissues [8]. In this way, the fluid deficiency was replaced, sufficient tissue perfusion was provided and the unwanted effects of anesthetics were tried to be removed. In this study, we found that there were no differences in administration of vasoactive drug, urinary and total volume of infusion of crystalloid. We maintained circulation stability through infusion volume and administration of vasoactive drug. We found urine volume was within the normal range, therefor circulation stability was approving and the unstable circulation abnormal results was excluded.

High lactate level have been associated with poor outcomes in the critically ill patients [9]. Lactate was initially introduced as an alkali. Its alkalinizing effect depended on its reutilization for glucose synthesis and its oxidative degradation to H_2_O and CO_2_, which was converted into bicarbonate. Abnormalities of lactate metabolism were very common in patients undergoing prolonged surgery. The metabolism of lactate depended on the kidney and liver, and as such, when the functions of these organs were compromised, there would be lactate accumulation [10]. Although we observed that lactate level was significantly higher in Group LR than in Group AR at the end of surgery, it was within the normal range. We hypothesized that liver function was limited to metabolise the lactate with biliary atresia patients and the lactate was accumulated, but the liver had a functional reserve and could metabolise lactate. The main organ that metabolizes lactate is liver, whereas acetate can be metabolized widely throughout the body and not mainly dependent on the liver. Its use in intravenous solutions is becoming popular, because it readily is converted to bicarbonate than lactate. In addition, acetate can be metabolized more quickly than lactate. This study showed no difference between the two groups in bicarbonate. In contrast, Kumar et al. used acetated crystalloid as an intraoperative fluid and the levels of bicarbonate and base excess showed an improved profile [11]. Increase in lactate level was commonly observed if the volume of liver was inadequate following major hepatectomy [12]. Sunil et al. found that the level of lactate in the lactated Ringer’s group was significantly higher than in the acetate solution group at the end of the operation [5]. In line with our result, acetated Ringer’s solution was found to be safer as compared to normal saline in protecting young children undergoing major surgery against the risk of increasing plasma chlorides and the subsequent metabolic acidosis [13].

This study showed that although serum chloride and serum sodium were significant higher at the end of surgery, the levels were within the normal range in both groups. The contribution of hyperchloremia toward persistent acidosis, however, did not seem to play a major role in our study population. Khan et al. found that lactate Ringer’s solution to prevent hyperchloremic metabolic acidosis [14]. We demonstrated that AR and LR may play a less role in electrolyte disorders including hyperchloremic and hypernatremia with fluid resuscitation. Concerns about intravenous hypotonic fluids have focused on potential neurological sequelae associated with severe hospital-induced hyponatremia [15]. Hyponatremic encephalopathy is the most crucial risk of acute hyponatremia and may result in permanent neurological damage or death. Hyponatremia was the common electrolyte disorder in children, affecting approximately 25% of hospitalized children and 30% of children in the postoperative period, most of which occurred after uncomplicated surgeries [16]. Stimulation of the antidiuretic hormone (ADH) may be due to hemodynamic causes such as hypovolemia and hypotension. Other factors that cause hemodynamic-independent non-osmotic ADH release include postoperative status, positive pressure ventilation, pain, nausea, vomiting and the use of narcotic medication [17]. As a result of decreased diuresis effect of kidney due to ADH over stimulation, fluid retention and related dilutional hyponatremia increase the risk of hyponatremic encephalopathy in pediatric patients in perioperative period [14].

The hyponatremia was not found in both groups. Hence, we could draw the conclusion that administration of acetate Ringer’s solution or lactate Ringer’s solution played a less role in leading hyponatremia. Intraoperative hypothermia was associated with numerous complications such as decreased drug metabolism, impairment of coagulation, and shivering [18]. In our study, we observed that the temperature was a signifcantly higher at postoperative 12 h in two groups. The high temperature was related to postoperative fever [19]. The inflammatory mechanisms accountable for postoperative fever have been the subject of a number of studies. Tissue damages alone resulted in the disruption of phospholipids from the cell membrane, and leaded to a cascade of prostaglandins and cytokines which ultimately leaded to a body temperature elevation. We used forced-air prewarming before anesthesia induction to prevent the development of hypothermia. Hypothermia was not observed in infant patients.

The incidence of hypoglycemia during induction of anesthesia was reported to be between 0 and 2.5%. In most of the children identified with hypoglycemia, an average of 10 h of fasting times were reported. Hypoglycemia was not observed in children who had drunk clear fluid up to 2 h before surgery [20]. Our study found that no hypoglycemia was recorded following fluid resuscitation in both groups. All infants patients received clear fluids containing 3 ml / kg glucose over the last 2 h before surgery, according to their weight calculated [21].

Limitations of the study included small sample size and lack of comparison of base excess. The data should have been collected up to postoperation 24 h. Postoperation coagulation function and hepatic function were not assessed and analyzed. Hemodynamics was not analyzed during operation. Only a single surgery was included in the study. Further research involving larger number of patients undergoing different surgeries is needed to know the acid base physiology in infant or neonatel patients.

In summary, resuscitation with AR and LR was associated with similar clinical improvement in infant with biliary atresia. Use of acetate Ringer’s solution reduced levels of lactate in comparison with LR. Hence, AR was to be considered as the fluid of choice owing to the clinical improvement with the Kasai hepatoportoenterostomy.

## Data Availability

Our raw data can be shared dy a public repository. https://pan.baidu.com/s/1N3oLoM3Fq9j6Lbf8dM2i8g

## Abbreviations

AR: Acetate Ringer’s solution
LR: Lactate Ringer’s solution
P_ET_CO_2_: End-tidal carbon dioxide partial pressure
PT: Prothrombin time
APTT: Activated partial thromboplastin time
ALT: Alanine transaminase
AST: Aspartate aminotransferase
ADH: Antidiuretic hormone
